# Generalized Net Model of the Foreign Object Principle and its Network Physiology Interpretations

**DOI:** 10.3389/fnetp.2022.873337

**Published:** 2022-04-20

**Authors:** Krassimir Atanassov, Galya Staneva, Tania Pencheva

**Affiliations:** ^1^ Department of Bioinformatics and Mathematical Modelling, Institute of Biophysics and Biomedical Engineering, Bulgarian Academy of Sciences, Sofia, Bulgaria; ^2^ Department of Lipid-protein Interactions, Institute of Biophysics and Biomedical Engineering, Bulgarian Academy of Sciences, Sofia, Bulgaria; ^3^ Department of QSAR and Molecular Modelling, Institute of Biophysics and Biomedical Engineering, Bulgarian Academy of Sciences, Sofia, Bulgaria

**Keywords:** foreign object principle, generalized net, model, index matrix, network physiology interpretation

## Abstract

The Foreign Object Principle has been introduced in a formalized form for first time. Proven as a suitable tool for modelling of parallel processes flowing in real time, the generalized nets have been used for an interpretation of the Foreign Object Principle. It is illustrated by some examples from network physiology.

## 1 Introduction

The human being is probably the most complex system ever known, no matter if one considers live or inanimate nature. Living organisms, and in particular—human beings, function as an autonomous complex of organ systems, organs, tissues, cell, etc. Meanwhile, the processes in the human body may be considered in some way in vertical axes—at different hierarchical levels of aforementioned organs, tissues, cell, etc., as well as—at the horizontal level, with all connections in the levels of organs, of tissues, of cells. The human body may be considered as a kind of continuous repetition of the principle of impact and reaction, from one hand—between the human body and the environment, and from the other hand—within the human body.

A plenty of models of human body are well known to the both scientific community and wider audience—graphical, descriptive ones, mathematical, physical, physico-chemical, biochemical ones, mechanistic, deterministic ones, etc. In this investigation, a model of the human body as an autonomous complex system, based on the apparatus of Generalized Nets [GNs, (Atanassov, 1991; Alexieva et al., 2007; Atanassov, 2007)] and reflecting the main principle of the impact and corresponding reaction, is going to be developed.

GNs have been proven as a powerful tool for modelling of parallel processes flow representation as well as of discrete event systems. The apparatus of GNs is equally well suited for modelling of large and complex systems, as well as for some simpler systems. One of the major strength of the GN apparatus is its ability to model random events and to predict the effects of complex interactions between these events. GN models might be used as a quick method for analysing and solving complex problems.

The concept of GNs as an extension of the Petri Nets (Petri, 1962; Starke, 1980) was defined in 1982, although the introducing paper (Atanassov, 1984) appeared 2 years later, in 1984. Very short remarks on a special case of GNs, called a minimal reduced GNs are presented later on, in Section 3.

The reduced GNs have the same modelling possibilities as the ordinary GNs, but they have only a part of the ordinary GN components. On the other hand, these nets have enough components to suitable to represent the objects and processes describing in the present paper.

Among the variety of filed of GN-models applications, such as economics, e-learning, artificial intelligence, data mining, biology and ecology, chemistry and economics, university administration processes and many others, GN-models have been developed in the area of medicine as well.

This investigation is a kind of long-waited continuation of the idea of the Foreign Object Principle and its GN interpretation, presented in (Atanassov, 2000; Sorsich et al., 2001). There the Foreign Object Principle was formulated as follows: each (abstract) system functions by taking (accepting) foreign objects from its environment and giving out objects to the environment. A formal definition of this Principle is given in the following Section 2. Developed in (Atanassov, 2000; Sorsich et al., 2001) GN-model serves the idea, that what is common for all the described processes in the human body is that a certain (material or not) foreign object is perceived, processed in one or another way, and as a result another (material or not) object is produced. It is being isolated in another (for instance the surrounding) environment and it appears in it in the form of “foreign object”. As such, an illustration of a new principle holding for the abstract theory of the systems (Mesarovic and Takahara, 1975; Mesarovic and Takahara, 1989), that can be conventionally named “The principle of the foreign object” has been presented.

## 2 Formal model

Let us denote the environment by *E*, the system—by *S*, and the temporal scale—by *T*. Let the impact functions be: *F*_*E*_—the impact of the environment over the system and *F*_*S*_—the impact of the system over the environment.

For the simplified form of the model, the system is presented in a fixed time-moment *t* ∈ *T*, in which the reaction of the system is defined by

$$R_{S}=F_{E}\left(E,S,t\right),$$

and a next time-moment *t*′, defined as

$$t^{\prime }=\tau_{E}\left(E,S,t\right),$$

in which the system reaction be changed.

Also, we need another fixed time-moment *t* ^″^ ∈ *T*, in which the reaction of the environment is defined by

$$R_{E}=F_{S}\left(S,E,t^{\prime \prime }\right),$$

and a next time-moment *t*′′′, defined as

$$t^{\prime \prime \prime }=\tau_{S}\left(S,E,t^{\prime \prime }\right),$$

in which an environment reaction will be changed.

**FIGURE 1:**
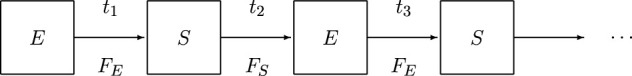
Simplified model.

Therefore, for given time-moments *t*_1_, *t*_2_, *t*_3_, … ∈ *T* we can write (see also Figure 1) *R*_*E*_ = *F*_*S*_[*S*, *E*, *τ*_*E*_(*E*, *S*, *t*_1_)] and *R*_*S*_ = *F*_*E*_[*E*, *S*, *τ*_*S*_(*S*, *E*, *t*_2_)].

**FIGURE 2:**
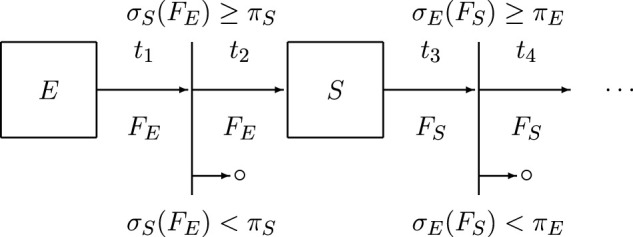
More complex model.

For the more complex model, we need functions *σ*_*S*_ and *σ*_*E*_ that give the values of the impact of the system or of the environment and determine whether these values are over or under the respective thresholds (i.e., *π*_*S*_ and *π*_*E*_). In this case, we can add additional time-moments for check the value of the impacts. Thus, the new model has the geometrical interpretation presented in Figure 2.

In Figure 2, the ◦-symbol stands for stop functioning of the signal.

## 3 Short Remarks on the Theory of the Generalized Nets

GNs are an extension as of the standard Petri nets, as well as of the rest of their extensions and modifications. GNs are defined in a way that is principally different from the ways of defining the other types of Petri nets (Atanassov, 1991; Atanassov, 2007).

When some of the GN-components are omitted, the GN is called a reduced GN. For the needs of the model below, we describe the modelled process as a reduced GNs.

**FIGURE 3:**
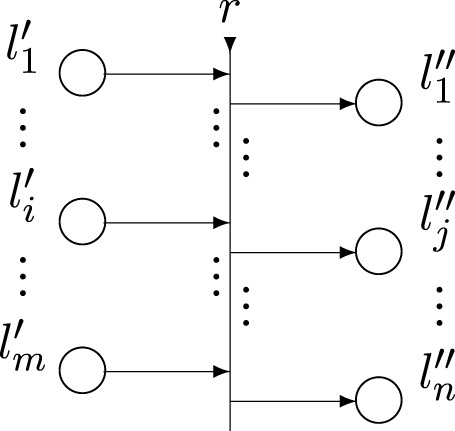
A generalized net transition.

Formally, every transition (Figure 3) is described by a seven-tuple, but for our aims, we use its following reduced form:

$$Z=\left\langle L^{\prime },L^{\prime \prime },r\right\rangle ,$$

where:

• *L*′ and *L* ^″^ are finite, non-empty sets of places (the transition’s input and output places, respectively); for the transition in Figure 3 these are $L^{\prime }=\left\{l_{1}^{\prime },l_{2}^{\prime },\ldots ,l_{m}^{\prime }\right\}$ and $L^{\prime \prime }=\left\{l_{1}^{\prime \prime },l_{2}^{\prime \prime },\ldots ,l_{n}^{\prime \prime }\right\};$

• *r* is the transition’s *condition* determining which tokens will pass (or *transfer*) from the transition’s inputs to its outputs; it has the form of an Index Matrix (IM; Atanassov, 2014):

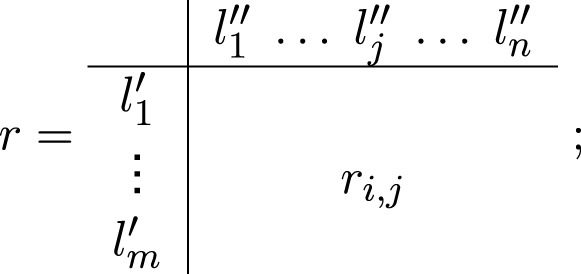
 *r*_*i*,*j*_ is the predicate that corresponds to the *i*-th input and *j*-th output place (1 ≤ *i* ≤ *m*, 1 ≤ *j* ≤ *n*). When its truth value is “*true*”, a token from the *i*-th input place transfers to the *j*-th output place; otherwise, this is not possible.

In general, the GN is defined as ordered four-tuple, but in the present case, it has the form

E=⟨A,K,X,Φ⟩,

where:

• *A* is a set of transitions;
• *K* is the set of the GN’s tokens.
• *X* is the set of all initial characteristics which the tokens can obtain on entering the net;
• Φ is the characteristic function that assigns new characteristics to every token when it makes the transfer from an input to an output place of a given transition.

The most important differences of the GNs in comparison to the Petri nets and other extensions of theirs are the following:

• Index matrices associated with the GN-transitions contain predicates that determine the directions of the token transfers;
• GN-token characteristics which contain the information for the processes related to the respective tokens and for the processes flowing in the net;
• Global time-scale to which the events in the net are referred to.

In the present paper, these predicates and token characteristics are given in not-formal form for reader’s facilitation.

Operations, relations and operators are defined over GNs (Atanassov, 1991; Atanassov, 2007).

## 4 Generalized Net Model

**FIGURE 4:**
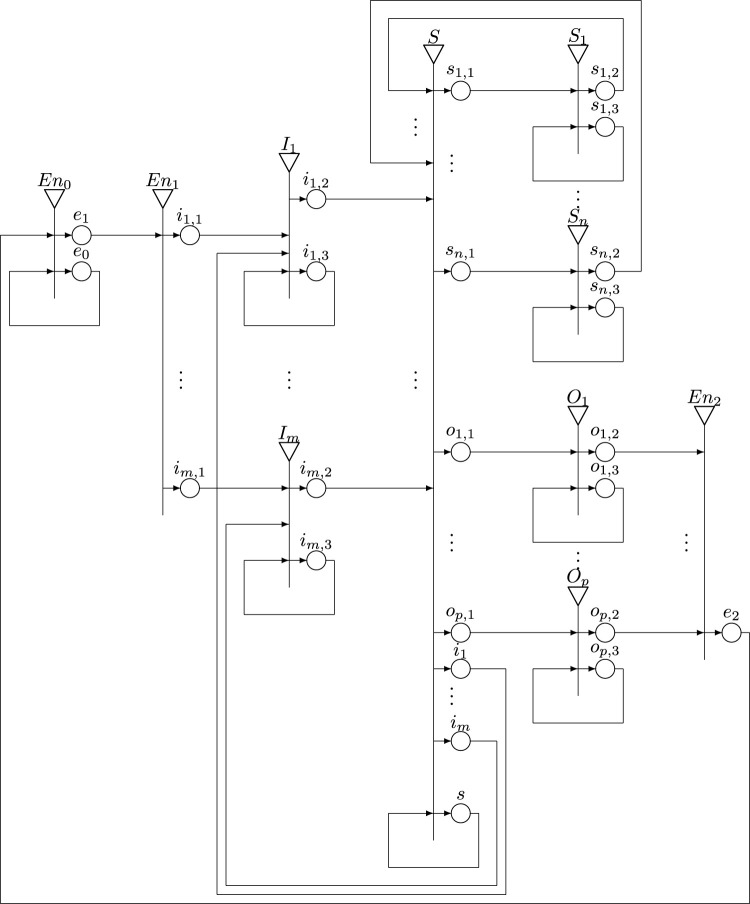
A generalized net model.

Figure 4 presents the generalized net model developed to describe in general the formal model from Section 2.

The GN contains *m* + *n* + *p* + 3 transitions, 4*m* + 3(*n* + *p* + 1) + 1 places and six types of tokens, that represent:

• ɛ*—information about the environment (the surroundings, the world around us, ambience, universe, etc.).
• *ɛ*—information about the impact from the environment to the system.
• *ω*—information about the system status.
• *φ*—information about the effector.
• *τ*—information about thresholds of the receptors/sensors.
• *κ*—the system signal for changing the value of the corresponding thresholds of the receptors/sensors.

The transitions have the following sense:

• *En*_0_, *En*_1_, *En*_2_—the environment (the surroundings, the world around us, ambience, universe, etc.).
• *I*_1_, *…*, *I*_*m*_—the impact from the environment to the system.
• *S*, *S*_1_, *…*, *S*_*n*_—the system.
• *O*_1_, *…*, *O*_*p*_—the effector.

The transition *En*_0_ has the following form:

$$En_{0}=\left\langle \left\{e_{0},e_{2}\right\},\left\{e_{0},e_{1}\right\},r_{En_{0}}\right\rangle ,$$

where
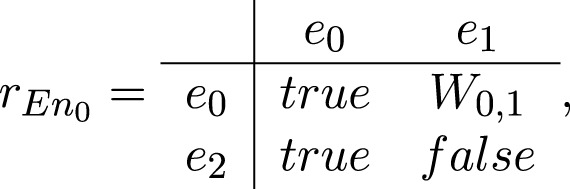


*W*_0,1_ = “there is an impact from the environment to the system”.

The token *ζ* from place *e*_2_ enters place *e*_0_ and unites with token ɛ* obtaining then a characteristic

“a result of the impact of the system over the environment”.

For each *k* = 1, *…*, *m*, the transition *En*_1_ has the following form:

$$En_{1}=\left\langle \left\{e_{1}\right\},\left\{i_{1,1},\ldots ,i_{m,1}\right\},r_{En_{1}}\right\rangle ,$$

where
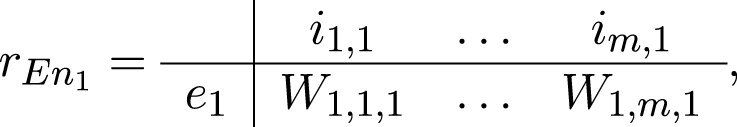
where *W*_1,*k*,1_ = “the current impact is registered by the *k*-th receptor/sensor”.

When *W*_1,*k*,1_ = *true*, token *ɛ* from place *e*_1_ enters place *i*_*k*,1_ without a new characteristic.

For each *k* = 1, *…*, *m*, the transition *I*_*k*_ has the form:

$$I_{k}=\left\langle \left\{i_{k,1},i_{k,3},i_{k}\right\},\left\{i_{k,2},i_{k,3}\right\},r_{I_{k}}\right\rangle ,$$

where
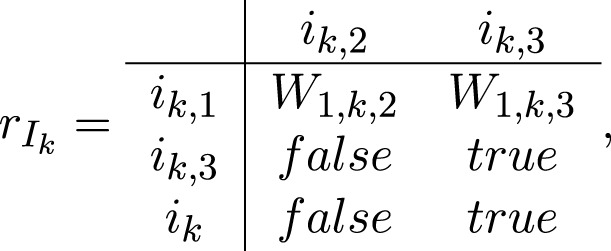
where

*W*_1,*k*,2_ = “the strength of the impact is greater than the threshold of the *k*-th receptor/sensor, determined as current characteristic of token *τ*_*k*_”, *W*_1,*k*,3_ = *¬W*_1,*k*,2_, and *¬P* is the negation of predicate *P*.

When *W*_1,*k*,2_ = *true*, token *ɛ* from place *i*_*k*,1_ enters place *i*_*k*,2_ without a new characteristic.

When *W*_1,*k*,3_ = *true*, token *ɛ* from place *i*_*k*,1_ enters place *i*_*k*,3_, unites with token *τ*_*k*_ and stops functioning. Token *τ*_*k*_ obtains then a new characteristic.

“an impact is registered by the *k*-th receptor sensor, parameters (time-moment, strength over or under the threshold)”.

Token *κ*_*k*_ from place *i*_*k*_ enters place *i*_*k*,3_, unites with token *τ*_*k*_ and obtains a new characteristic

“*new value of the threshold of the* *k* *-th receptor/sensor”*.

For *k* = 1, *…*, *n*, the transition *S* has the following form:

$$S=\left\langle \left\{i_{1,2},\ldots ,i_{m,2},s_{1,2},\ldots ,s_{n,2},s\right\},\right.$$

$$\left.\left\{s_{1,1},\ldots ,s_{n,1},o_{1,1},\ldots ,o_{p,1},i_{1},\ldots i_{m},s\right\},r_{S}\right\rangle ,$$

where
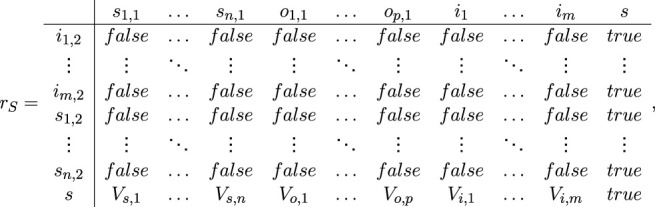
and for *k* = 1, *…*, *n*:

*V*_*s*,*k*_ = “the registered impact must be processed by the *k*-th system component”, for *k* = 1, *…*, *p*:

*V*_*o*,*k*_ = “a new impact of the system over the environment may occur through the *k*-th effector”, for *k* = 1, *…*, *m*:

*V*_*i*,*k*_ = “the value of the threshold of the *k*-th receptor/sensor must be changed”.

The *ɛ* tokens (one or more) from places *i*_1,2_, *…*, *i*_*m*,2_ enter(s) place *s*, where it (they) unite(s) with token *ω* and obtain(s) a characteristic

“registration of the impact, parameters”.

The *σ*-tokens (one or more) from places *s*_1,2_, *…*, *s*_*n*,2_ enter(s) place *s*, where it (they) unite(s) with token *ω* and obtain(s) a characteristic

“reaction of the respective part of the system to the corresponding impact”.

When for some *k* = 1, *…*, *n* the predicate *V*_*s*,*k*_ = *true*, token *ω* splits to two or more tokens: the same token *ω* that continues staying in place *s* without a new characteristic, and to one or more *σ*-tokens that enter(s) respective *s*-place with a characteristic.

“the current impact must be processed by the respective part of the system”.

When for some *k* = 1, *…*, *p* the predicate *V*_*o*,*k*_ = *true*, token *ω* splits to two or more Tokens: the same token *ω* that continues staying in place *s* without a new characteristic, and to one or more *π*-tokens that enter(s) respective *o*-place with a characteristic

“the prepared by the system reaction must be directed to the respective effector”.

When for some *k* = 1, *…*, *m* the predicate *V*_*i*,*k*_ = *true*, token *ω* splits to two or more tokens: the same token *ω* that continues staying in place *s* without a new characteristic, and to one or more *κ*-tokens that enter(s) respective *i*-place with a characteristic

“new value of the threshold of the respective receptor/sensor”.

For each *k* = 1, *…*, *n*, the transition *S*_*k*_ has the form

$$S_{k}=\left\langle \left\{s_{k,1},s_{k,3}\right\},\left\{s_{k,2},s_{k,3}\right\},r_{S_{k}}\right\rangle ,$$

where
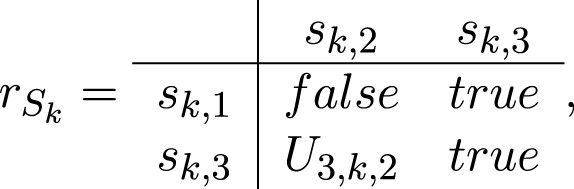
where

*U*_3,*k*,2_ = “the *k*-th system component is ready with generated response to the registered impact”.

When *U*_3,*k*,2_ = *true*, token *σ* splits to two tokens: the same token *σ* that continues staying in place *s*_*k*,3_ without a new characteristic, and token *σ*′ that enters place *s*_*k*,2_ with a characteristic

*“response to the registered impact, parameters”.*

For each *k* = 1, *…*, *p*, the transition *O*_*k*_ has the form

$$O_{k}=\left\langle \left\{o_{k,1},o_{k,3}\right\},\left\{o_{k,2},o_{k,3}\right\},r_{O_{k}}\right\rangle ,$$

where
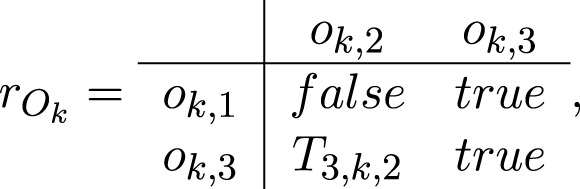
where

*T*_3,*k*,2_ = “there is a ready system reaction for the *k*-th effector”.

When *T*_3,*k*,2_ = *true*, token *φ* splits to two tokens: the same token *φ* that continues staying in place *o*_*k*,3_ without a new characteristic, and token *φ*′ that enters place *o*_*k*,2_ with a characteristic

“system reaction for the k-th effector, parameters”.

Transition *En*_2_ has the form

$$En_{2}=\left\langle \left\{o_{1,2},\ldots ,o_{p,2}\right\},\left\{e_{2}\right\},r_{En_{2}}\right\rangle ,$$

where
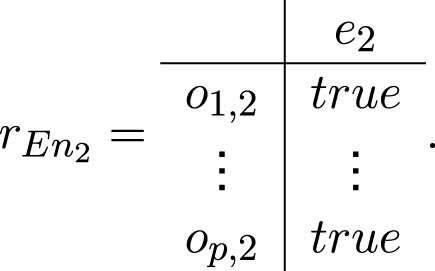


All tokens from places *o*_1,2_, *…*, *o*_*p*,2_ unite in one token *ζ* that enters place *e*_2_ with a characteristic

“total system reaction, parameters”.

## 5 Examples

In general, a living organism cannot be separated from its surroundings since it is in of a continuous need of oxygen, water and food. Organisms themselves, and human being in particular, are in a continuous exchange of matter and energy with their environment. The interaction between organisms and the environment has two components: input, describing what enters the system from the outside or as a reaction of the system (the *i*-places in the GN model from Figure 4, and output, describing what leaves the system for the environment (the *o*-places in the GN model). In order to be able to speak about the inside and the outside of a system, it is needed to be defined a kind of boundary between the system itself and its environment. For example, the skin might play such a role of a physical boundary for living systems (in the GN model it corresponds to an *I*-transition).

Let us consider, as an example, how the GN model describes the reaction of human body, and in particular - of the central nervous system, to pain, caused by a pin prick of a finger. The sharp pin itself might be considered as an input from environment (transition *En*_0_). The first reaction of the system (reflected by the one of the *I*-transitions) is the activation of the pain receptors of the skin. The signals are then passed through the spinal cord, which corresponds to the transition *S* in the GN model. Finally, the response of the system will be a withdrawal of the arm caused by a contraction of the biceps, which is reflected by a *S*-transitions of the GN model.

Another example of human pain reaction induced by a direct external or internal (also caused by environment impact) injuries of human body is the chronic pain. After an acute short lasting pain, opposite to the chronic pain, the human body reacts to the brain signal by fighting or escaping the source of pain through complex adaptive responses (*S*-transitions) such as increased heart rate, respiratory rate, muscle contractions and others. At chronic pain, human is not able to escape and the neural pathways in the brain and central nervous system systematically change (*I*-transitions). The long lasting pain induces a variety of physiological and psychological responses such as insomnia, exhausted physical and brain fatigue which in turn reduces the pain threshold (*I*-transitions). Furthermore, such patients develop hypersensitivity experiencing more pain than they should to external physical stimulus such as mild push on the skin, noises, smells, lights and others. Examples of such medical conditions are long lasting migraine headache and fibromyalgia. Two different ways to interrupt the vicious pain circle could be used to increase the pain threshold: 1) by more and more interactions between the system (patient) and the environment (distraction activities); 2) by using pharmaceuticals (opioids, anti-inflammatory or antidepressant drugs). In the case of short lasting acute pain, the system and the environment interact only one time under the external stimulus whereas in the chronic pain case, the system and the environment interact multiple times as the system adapts continuously to the environment stimuli.

In general, the output is quite different from the input. The system is not just passively attending, but plays an active role. For example, the food, drink and oxygen are taken by the living organisms (through the transition *En*_0_). Further, they are processed through different sensors for taste, smell, touch, etc. [through an *I*-transition(s)], provoke different reactions and processes in the body (described in the transition *S*) and quit the body as urine, excrements and carbon dioxide [through an *O*-transition(s)].

For many processes in the human body almost no one does not know how they happen. Doctors may observe that if they give a patient a particular medicine (input, transition *En*_0_), the patient will react in a certain way (some *o*-place), e.g., by producing more urine. However, in most cases even the doctors have just a general idea about the particular mechanisms which lead from the cause to the effect. Obviously, the medicine triggers a complex chain of interconnected reactions, involving different organs and parts of the body (some of transitions *S*_1_, *…*, *S*_*n*_), but the only thing that can be clearly established is the final result.

There is a potential for further extension of developed GN model in different directions. One of them is if one looks more closely at the environment of a given system, he can consider it as a number of systems interacting with their environments. For example, the environment of a person usually is a kind of community of other persons. Such groups of interacting people may form a family, a company, or a city. Their mutual interactions may lead to “glue” the components into a whole. If these parts did not interact, the whole would not be more than a sum of its components. But due to their interactions there is something to be added. With respect to the whole, the parts might be considered as subsystems. With respect to the parts, the whole might be considered as a supersystem. If now one consider a collection of such systems which interact with each other, that collection could again be seen as a system. If one looks at the supersystem as a whole, he should not need be aware of all its parts. He can again just look at its total input and total output without worrying which part of the input goes to which subsystem.

## 6 Conclusion

Presented here is a GN model of the functioning of a system, from the point of view of the Foreign Object Principle. It is illustrated with examples related to the human body as an autonomous complex system. The GN apparatus allows at reflection of the main principle of the impact and corresponding reaction. The developed GN model also allows to be shown the continuous repetition of aforementioned principle, from one hand—between the human body and the environment and vice versa, and from the other hand—within the human body. As it has been shown in the developed here reduced GN-model, the GN apparatus allows at accounting the temporal component, which makes enable a bridge to be thrown in the terms of Network Physiology, namely from accounting the temporal dynamics in such a complex system as human body to the synchronization and principles of integration in Networks of Physiological Systems.

Since the human body itself is as a universe, the present model might be extended in many directions, i.e., by more detailed description of the processes, related to place *o* and its token *ω*.

In the future, the GN-model can be extended with intuitionistic fuzzy evaluations e.g., (Atanassov, 2017) of the input objects (signals) from the environment, of the perception thresholds, of the output objects (signals) generated by the system to the environment and the thresholds allowing their transmission. In addition, the present research is an illustration of the use of the GN-approach for modelling of processes that relate to the area of Data Mining [cf. (Zoteva and Krawczak, 2017; Atanassov, 2020))].

## Data Availability

The original contributions presented in the study are included in the article/Supplementary Material, further inquiries can be directed to the corresponding author.
